# High Curie temperature ferromagnetic structures of (Sb_2_Te_3_)_1−x_(MnSb_2_Te_4_)_x_ with x = 0.7–0.8

**DOI:** 10.1038/s41598-023-34585-y

**Published:** 2023-05-06

**Authors:** Ido Levy, Candice Forrester, Xiaxin Ding, Christophe Testelin, Lia Krusin-Elbaum, Maria C. Tamargo

**Affiliations:** 1 Department of Chemistry, The City College of New York, New York, NY 10031 USA; 2 Ph.D. Program in Chemistry, The Graduate Center of the City University of New York, New York, NY 10016 USA; 3 Department of Physics, The City College of New York, New York, NY 10031 USA; 4 Sorbonne Université, CNRS, Institut des NanoSciences de Paris, 75005 Paris, France; 5 Ph.D. Program in Physics, The Graduate Center of the City University of New York, New York, NY 10016 USA

**Keywords:** Materials science, Nanoscience and technology, Physics

## Abstract

Magnetic topological materials are promising for realizing novel quantum physical phenomena. Among these, bulk Mn-rich MnSb_2_Te_4_ is ferromagnetic due to Mn_Sb_ antisites and has relatively high Curie temperatures (T_C_), which is attractive for technological applications. We have previously reported the growth of materials with the formula (Sb_2_Te_3_)_1−x_(MnSb_2_Te_4_)_x_, where x varies between 0 and 1. Here we report on their magnetic and transport properties. We show that the samples are divided into three groups based on the value of x (or the percent septuple layers within the crystals) and their corresponding T_C_ values. Samples that contain x < 0.7 or x > 0.9 have a single T_C_ value of 15–20 K and 20–30 K, respectively, while samples with 0.7 < x < 0.8 exhibit two T_C_ values, one (T_C1_) at ~ 25 K and the second (T_C2_) reaching values above 80 K, almost twice as high as any reported value to date for these types of materials. Structural analysis shows that samples with 0.7 < x < 0.8 have large regions of only SLs, while other regions have isolated QLs embedded within the SL lattice. We propose that the SL regions give rise to a T_C1_ of ~ 20 to 30 K, and regions with isolated QLs are responsible for the higher T_C2_ values. Our results have important implications for the design of magnetic topological materials having enhanced properties.

## Introduction

Magnetic topological materials, such as topological insulators (TIs) and Weyl semimetals, are being intensively investigated due to the prediction and recent observation of the quantum anomalous Hall Effect (QAHE)^1^, axion insulator state^2^ and other exotic quantum phenomena, as well as ensuing potential applications in spintronics^3^ and quantum computing^4^. The first observation of QAHE in TI systems was reported in a Cr doped (Bi, Sb)_2_Te_3_, however due to a high defect density produced by the Cr impurity atoms the QAHE was only observed at sub-Kelvin temperatures^5–9^. A class of intrinsic magnetic TIs were discovered when Mn was added to Bi and Te (or Se) during crystal growth. The addition of sufficient Mn results in the formation of MnBi_2_Te_4_^10^ (or MnBi_2_Se_4_^11^) septuple layers (SLs), instead of the well-known quintuple layer (QL) structure of the non-magnetic TIs (e.g., Bi_2_Se_3_ and others). The formation of SLs is observed in bulk and epitaxial growth conditions, where, depending on the amount of Mn incorporated, the crystals self-assemble into mixtures of SLs and QLs. This discovery suggested the likelihood of fewer structural defects in these materials compared to Cr doped TIs, and the possibility to observe these physical phenomena at higher temperatures.

While a single SL of MnBi_2_Te_4_ is ferromagnetic (FM), it has been found that stacked SLs couple antiferromagnetically to each other^12^, which prevents the achievement of the QAHE. It has been shown that one way to stabilize a FM phase in such a system is to separate the magnetic SLs with non-magnetic QLs. This separation reduces the magnetic coupling between SLs, allowing FM alignment^13^. However, in the case of MnSb_2_Te_4_, this separation of the SLs with non-magnetic layers is not needed to achieve FM material^14,15^. Instead, it has been shown that the presence of Mn_Sb_ antisites can also induce the FM phase. Early works of growth combining Sb and Te with a small amount of Mn (1.7%), report FM materials with a Curie temperature (T_C_) 17 K^16^, likely emanating from single SLs embedded within the QLs. Recently, the bulk MnSb_2_Te_4_ system showed further increase of the T_C_ up to 33 K^17^. The highest reported T_C_ value to date for these materials was reported in Mn-rich MnSb_2_Te_4_ systems with T_C_ of 45–50 K^18^. In spite of these promising numbers, the MnSb_2_Te_4_ system has been far less investigated than MnBi_2_Te_4_ in the literature, and an understanding of the origin and control of its magnetic properties is still lacking. Furthermore, recent theoretical and experimental reports suggest that ferromagnetic MnSb_2_Te_4_ is a Weyl semimetal^19^, while others suggest that types and levels of magnetic disorder in Mn-rich samples modify the ensuing band structure, rendering the material a topological insulator^18^. The overwhelming interest in these materials along with the relatively limited understanding of the structural-property relationship, implies that investigations of the magnetic properties as they relate to the materials structural parameters are warranted and that they may provide a more directed approach to the crystal growth of the materials with the desired magnetic properties, while perhaps revealing new exotic physical phenomena that may surpass current achievements.

We recently performed a detailed study of the growth of (Sb_2_Te_3_)_1−x_(MnSb_2_Te_4_)_x_ structures (with x typically given as the percent septuple layer or %SL) by self-assembly in molecular beam epitaxy (MBE)^20^. We showed that the composition of the structures given by the value of x (or the %SLs) could be varied by controlling the relative Mn to Sb fluxes. Crystal structures spanning the full composition range, between Sb_2_Te_3_ (x = 0) to MnSb_2_Te_4_ (x = 1), could be grown. We also saw that our samples with only a few SLs were all FM. Detailed analysis of the high-resolution transmission electron microscopy (TEM) images showed that Mn_Sb_ and Sb_Mn_ antisite defects were present in our samples^20^.

In this work, we report the investigations of the magnetic properties of those samples with emphasis on their observed T_C_ values and the relationship of these values to the structural details of these materials. We use temperature dependent Hall resistance (R_xy_) plots at near-zero external magnetic fields to extract the T_C_ of the samples. The results show that the samples can be separated into three groups according to the behavior of their T_C_ values, which depend on the value of x (i.e., the %SL) in the structure. Group 1 contains the samples with less than 70% SLs, group 2 the samples with 70–80% SLs and group 3 the samples with more than 90% SLs. The R_xy_ plots for the samples from groups 1 and 3 can be described by a single T_C_ value, with T_C_ values of 15–20 K for the samples in group 1 and 20–30 K for the samples in group 3. By contrast, the R_xy_ plots of the samples in group 2 present a different shape, with a T_C_ value as high as 80 K, significantly higher than the highest reported value to date for this type of materials^18^. To confirm the high T_C_ value in the materials, field dependent R_xy_ measurements at high temperatures show a hysteresis loop around zero field at temperatures as high as 80 K in samples from this group. Further corroboration was obtained from temperature dependent magnetization measurements using a SQUID magnetometer. We also investigated the carrier density dependence on the %SLs of the samples. Our results indicate that under our MBE growth conditions, the QLs in our mixed SL:QL structures are likely to contain large Mn excess, making them very highly p-type doped electrically, while the SLs have a lower carrier density than the QLs. These findings are essential for the directed design and growth of TIs with the desired optimum magnetic and electrical properties.

## Results

As previously reported, Ref.^20^ samples of (Sb_2_Te_3_)_1−x_(MnSb_2_Te_4_)_x_ grown by MBE with x values ranging between x = 0 and x = 1, were obtained by varying the ratio of the Mn flux relative to the total Mn plus Sb flux (Mn flux ratio) used during growth. The fluxes were measured by monitoring the beam equivalent pressure (BEP) measured by an ion gauge at the position of the substrate prior to each growth. Details of the MBE growth conditions are given in the Supporting Information (Sect. 1).

**Figure 1:**
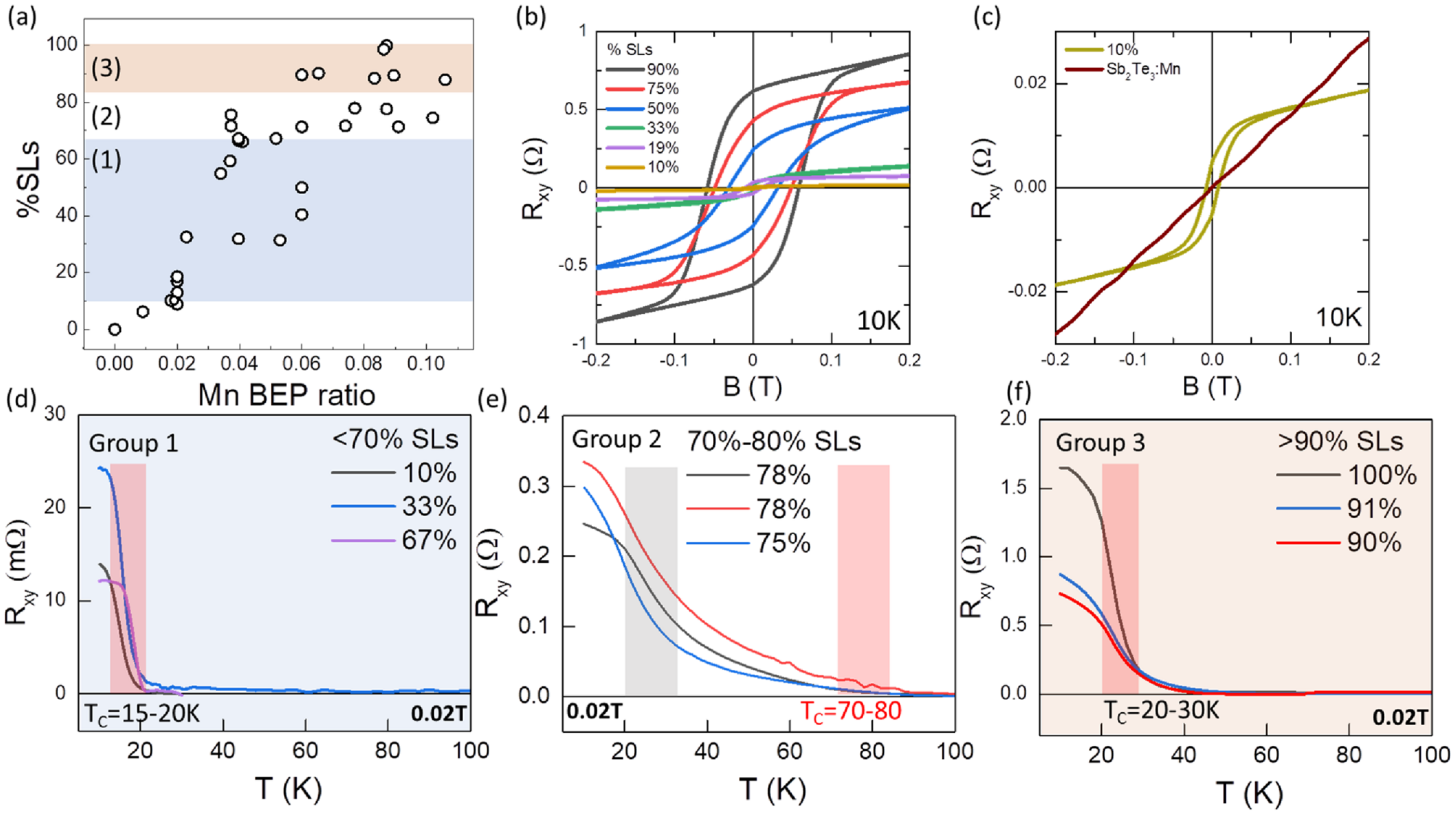
Magnetic properties of the (Sb_2_Te_3_)_1−x_(MnSb_2_Te_4_)_x_ samples. (**a**) Distribution of the SL fraction (x) of all samples as a function of the Mn BEP ratio adapted from ref. 20. The three groups of samples with different Curie temperature behavior are indicated by shading of their areas blue, white and red for samples with x < 0.7 (group1), 0.7 < x < 0.8 (group 2) and x > 0.9 (group 3), respectively. (**b**) Hysteresis loop of the Hall resistance (R_xy_) as a function of the magnetic field, B, of a selection of samples with x values ranging from 0.1 to 0.9 measured at T = 10 K. (**c**) Hall resistance (R_xy_) measurements of a sample with x = 0.1 (10% SLs) as well as a Mn doped all-QL Sb_2_Te_3_ sample (x = 0). The latter was grown with less than 0.01 Mn BEP ratio. (**d**–**f**) Temperature dependent R_xy_ plots under low magnetic field of 0.02 T for representative samples from the three groups identified in (**a**): (**d**) group 1, (**e**) group 2, and (**f**) group 3.

Figure 1a (adapted from Ref.^20^) shows a plot of the values of x obtained for the samples as a function of the Mn BEP ratio used during growth. The values of x for each sample (or the %SLs) were calculated from a previously determined calibration of the relationship of the HR-XRD peak position of the (0015) peak of Sb_2_Te_3_ as it shifts to the (0021) peak of MnSb_2_Te_4_, compared to the %SLs extracted from TEM images measured for several samples. In that study^20^, we concluded that the Mn incorporates in two ways into the grown samples: as a structural element in the crystal to form SL and as a dopant impurity in the QLs and the SLs. That conclusion was supported by the percent of Mn measured for several samples using energy dispersive X-ray spectroscopy (EDS), which indicated that higher levels of Mn than the levels expected based on stoichiometric SLs were observed^20^. Transmission electron microscopy (TEM) analysis also revealed that the excess Mn impurity atoms incorporate mostly in Sb sites (Mn_Sb_), while some Sb atoms were also incorporated at Mn sites (Sb_Mn_) as antisite defects^20^.

We found that all the samples that contain at least a few SLs are ferromagnetic (FM). Figure 1b shows field (B) dependent Hall resistance (R_xy_) measurements of a selection of the grown samples with varying %SLs, measured at T = 10 K. The data show a hysteresis loop around zero-field for all the samples, a sign of ferromagnetism. An expanded plot of a sample with only 10% SLs (x = 0.1), seen in Fig. 1c, clearly shows the hysteresis even in that sample. Figure 1c also shows, for comparison, the Hall resistance data for a Mn containing Sb_2_Te_3_ sample, grown with a very low Mn BEP ratio. At these low Mn fluxes the sample does not have any SLs and contains no detectable levels of Mn in the XRD plot. We expect Mn levels below 0.1% and x = 0. This sample shows a linear plot with no hysteresis, suggesting the absence of ferromagnetism at low Mn BEP ratios, when no SLs are formed, and Mn behaves only as an electrical dopant^21^.

In order to determine the Curie temperature (T_C_) of the samples, temperature dependent R_xy_ measurements were performed on all the samples. The measurements were made under a small applied magnetic field of 0.02 T, and are shown in Fig. 1d–f. Three different behaviors of the T_C_ were observed, which depended on the value of %SLs. Based on these behaviors, the samples were separated into three groups: group 1 for samples with less than 70% SLs, group 2 for samples with 70–80% SLs and group 3 for samples with more than 90% SLs, as shown in Fig. 1a by the blue, white, and red shaded areas, respectively. A selection of R_xy_ plots from each group is shown in Fig. 1d–f. From the shape of the plots in group 1, shown in Fig. 1d, a sharp decrease in R_xy_ as a function of temperature is observed. Assuming T_C_ is given by the region of steepest slope in the R_xy_ curve, all the samples in group 1 have T_C_ values ranging between 15 and 20 K as indicated by the red shaded column. In Fig. 1f, which presents representative data of samples in group 3, a similar sharp decrease in R_xy_ as a function of temperature is also seen, but with higher T_C_ values, ranging between 20 and 30 K as indicated by the red shaded column in that plot. A small tail extending to 40 K is also observed in the R_xy_ plots of the group 3 samples suggesting a T_C_ as high as 40 K. By contrast, the T_C_ plots for the samples in group 2, shown in Fig. 1e, exhibit a different behavior. Instead of a sharp drop in resistance as the temperature increased, featuring a single slope, the plots for this region show two distinct slopes, an initial strong downward slope pointing to about 20–30 K, indicated by the gray shaded column in Fig. 1e, and a second more gradual slope that persists to much higher temperature, as high as 70–80 K for some of the samples. This is highlighted by the red shaded column in Fig. 1e. We suggest that our structures contain two components each with a different T_C_ value: T_C1_ indicated by the gray shaded area in the plot, and T_C2_ indicated by the red shaded area. Further evidence for the presence of two T_C_ components and a rationale for this interpretation is provided in the text that follows.

**Figure 2:**
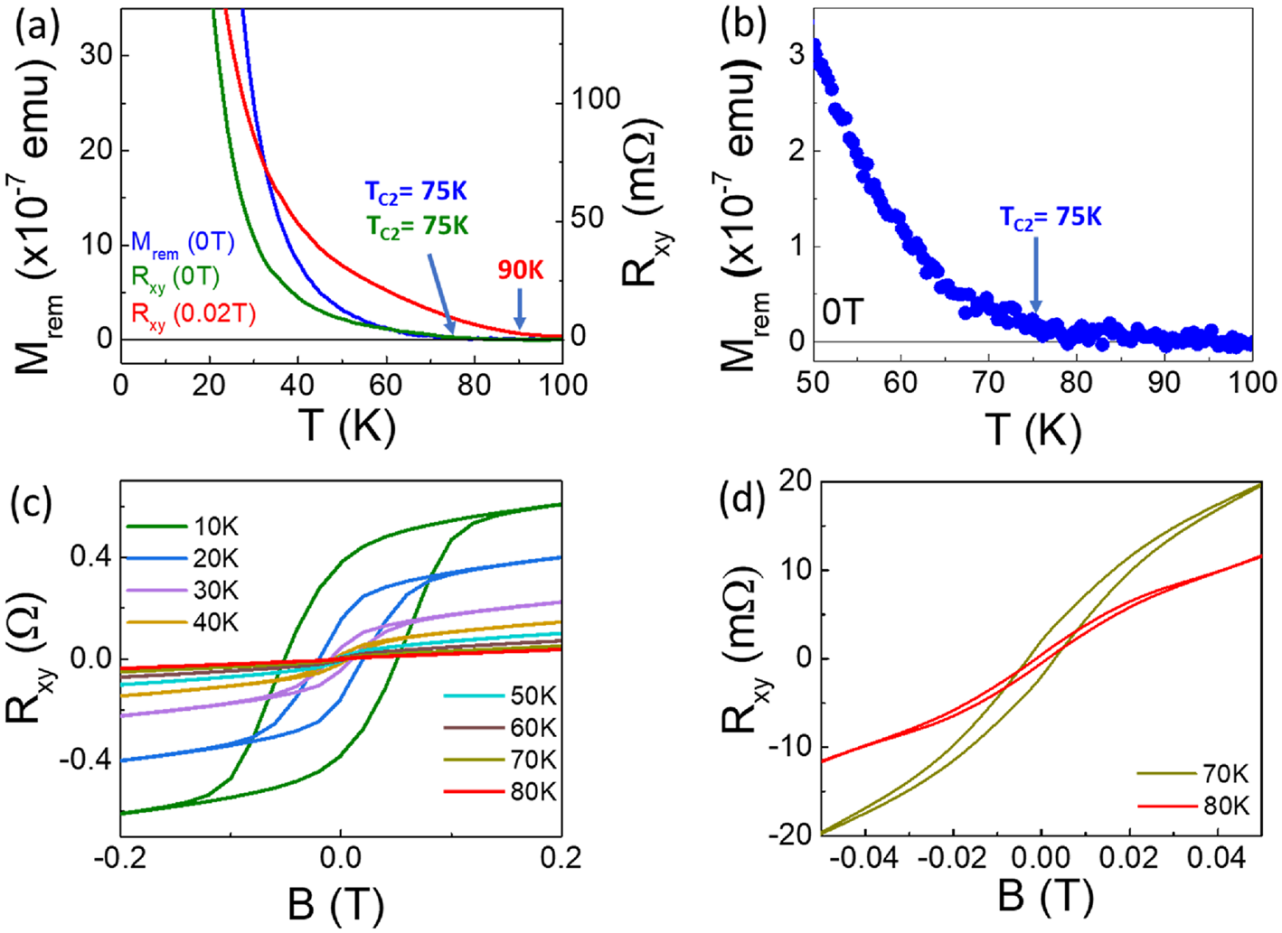
Magnetic measurements of a sample containing 75% SLs. (**a**) Temperature dependent Hall resistance (R_xy_) plots taken at 0.02 T and 0 T, as well as the remanent magnetization (M_rem_) taken at 0 T using a SQUID magnetometer. (**b**) Magnification of the M_rem_ plot to clearly identify its T_C2_ value. (**c**) Hysteresis loop of the Hall resistance (R_xy_) as a function of the magnetic field, B, measured at temperatures between 10 and 80 K for the sample with 75% SLs. (**d**) Magnification of the plots of 70 K and 80 K from (**c**) to better see their hysteresis loops.

To further demonstrate the presence of the high T_C_ value (T_C2_) of 70–80 K we compared the temperature dependent Hall resistance (R_xy_) plot measured at a field of 0.02 T to R_xy_ plots made at zero field, and to remanent magnetization (M_rem_) measurements made at zero field using a SQUID magnetometer, for a sample consisting of (Sb_2_Te_3_)_0.25_(MnSb_2_Te_4_)_0.75_. The data are shown in Fig. 2a. The measurements were performed as follows: for the R_xy_ plot under 0.02 T, the sample was cooled down and, at specific temperatures, the field was turned up to 0.02 T and the resistance was measured. This is the way the R_xy_ plots of Fig. 1d–f were all carried out. For the zero-field (0 T) R_xy_ and the remanent magnetization (M_rem_) measurements, the samples were cooled down under a field of 0.2 T for R_xy_ and 0.5 T for M_rem_ then heated up and measured under zero-field. The data are shown in Fig. 2a. Two different slopes are visible in the magnetization measurements, consistent with the R_xy_ measurements. However, while the R_xy_ (0 T) and the M_rem_ plots are very similar in shape, the R_xy_ at 0.02 T has a stronger signal of the high T_C_ component. The R_xy_ (0.02 T) plots also present a somewhat higher T_C2_ value of about 90 K, while the R_xy_(0 T) and M_rem_ give a T_C2_ value of about 75–80 K. This difference is probably due to an enhanced alignment of the magnetic spins under the small (0.02 T) field. Figure 2b is an expanded view of the higher temperatures of the M_rem_ plot that clearly shows a T_C2_ value of 75 K. Additional evidence that the FM phase in the sample persists at the higher temperatures was obtained from field dependent R_xy_ measurements performed at different temperatures (Fig. 2c). These measurements show a clear hysteresis loop up to 80 K (Fig. 2d), similar to the value extracted from the temperature dependent M_rem_ measurement, supporting the validity of the high T_C_ value (T_C2_) extracted from the temperature dependent R_xy_ plot. R_xy_ measurements done at 85 K (not shown here) show no hysteresis, consistent with the value determined from the 0 T measurements.

To analyze the lower temperature regime and support the presence of the proposed T_C1_ value, we performed Arrott plots^22^ of the magnetization data following mean-field theory behavior. From these plots (Supporting Information Fig. S1b), a low Tc value of ~ 23 K is clearly obtained for the sample of Fig. 2a, consistent with the T_C_ values of the Arrott plots of samples in groups 1 and 3 (Supporting Information Fig. S1a,c). We refer to this as T_C1_, while the second T_C_ component (T_C2_) is clearly evident from the R_xy_ and magnetization data and the high temperature hysteresis of Fig. 2c,d. Derivative plots of the temperature dependent Hall resistance (R_xy_) for two samples from group 2 (Supporting Information Fig. S2) also support the presence of two T_C_ components in samples having 70–80%SLs (group 2).

**Figure 3:**
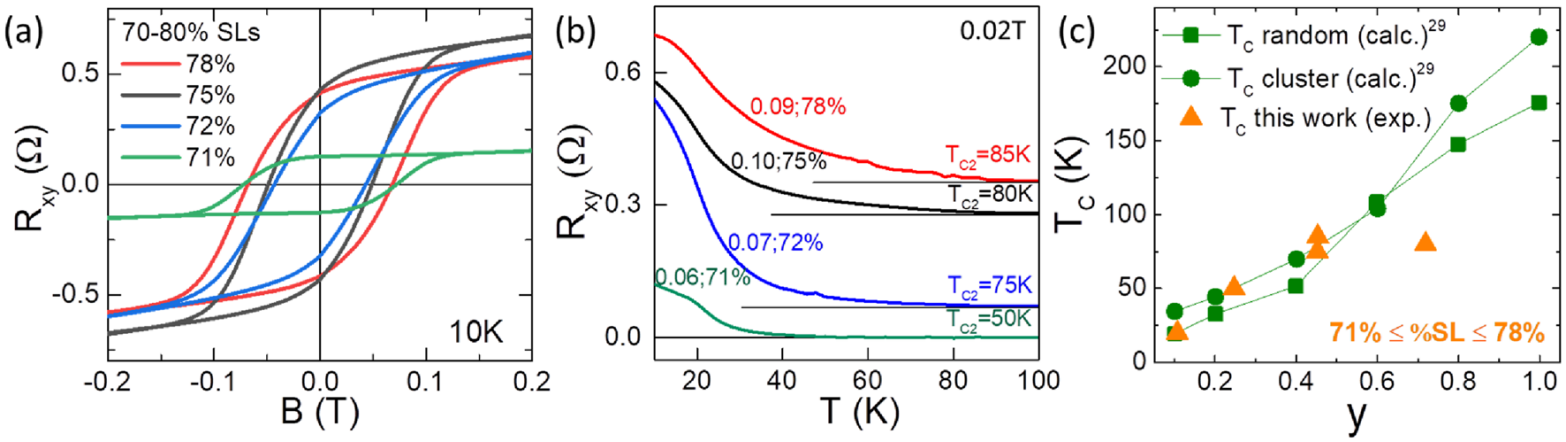
Measurements of a selection of samples from group 2 grown with Mn BEP ratios of 0.06–0.10 and having 70–80% SLs. (**a**) Hall resistance (R_xy_) as a function of the magnetic field B measured at 10 K. (**b**) Temperature dependent R_xy_ plots taken at 0.02 T, offset to clearly show their trends. The values of the Mn BEP ratio and the %SLs are given, as well as the T_C2_ values, color coded, for each plot. The black line is the 0Ω line for each plot. (**c**) Plot of Curie temperature as a function of the value of y in QLs of composition Sb_2−y_Mn_y_Te_3_ calculated for our samples as described in the text. Theoretically predicted values assuming two different types of Mn distributions: random and cluster, adapted from Vergniory et al.^29^ are given for comparison.

Careful observation of the data from group 2 in the plot of Fig. 1a shows that samples with x = 0.7–0.8 were formed under a large range of Mn flux ratios, given by the BEP ratios of 0.04–0.10. This implies that the Mn content in that set of samples varies even though the %SLs are all very similar. EDS measurements of the Mn fraction (χ_Mn_) in these samples, summarized in Table 1, confirm the increase in χ_Mn_ as the Mn BEP ratio increases. We measured the temperature dependent Hall resistance for four samples in this group that were grown with different Mn BEP ratios. Figure 3 shows the field dependent R_xy_ hysteresis plots at 10 K (Fig. 3a) and the temperature dependence of the R_xy_ (T_C_ plot) at 0.02 T (Fig. 3b) for the four samples. The field dependent R_xy_ curves (Fig. 3a) show a hysteresis around zero-field for all the samples, confirming the samples are all ferromagnetic at 10 K. In the temperature dependent measurements (Fig. 3b), the T_C2_ component in the samples becomes weaker, and the T_C2_ value of the samples decreases as the Mn BEP ratio decreases. Table 1 summarizes the relationship between the compositional details of the samples and their T_C2_ values. From these results we conclude that higher Mn content gives rise to higher values for T_C2_ (increasing from 20 to 85 K). The data show that besides having the appropriate 70–80% SLs, it is important that higher Mn BEP ratios are used during growth in order to obtain the higher temperature T_C2_ components.

**Table 1:** Compositional details and Curie temperatures of (Sb_2_Te_3_)_1−x_(MnSb_2_Te_4_)_x_ samples with 70–80%SLs. Parameter y for QL composition Sb_2−y_Mn_y_Te_3_ is introduced and described in the discussion section.

x	Mn BEP ratio	χ_*Mn*_ (%)	T_C_ (K)	C.C. (10^20^ cm^−3^)	y
0.76	0.04	13.3	20	–	0.107
0.71	0.06	14.9	50	3.8	0.247
0.72	0.07	18.2	75	4.3	0.452
0.78	0.09	18.8	85	2.4	0.453
0.75	0.10	22.6	80	3.9	0.719

**Figure 4:**
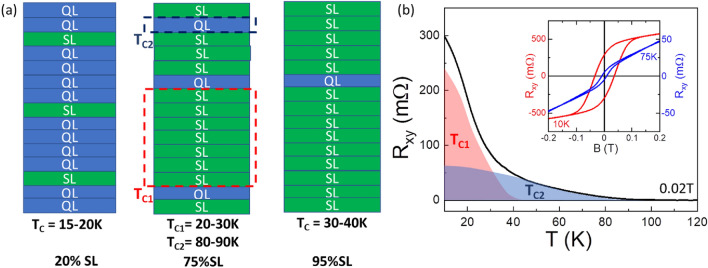
Structural characteristics of the (Sb_2_Te_3_)_1−x_(MnSb_2_Te_4_)_x_ samples that may lead to two T_C_ components. (**a**) Illustration of the layers of samples from the three groups defined in Fig. 1a, QLs and SLs are marked in blue and green respectively. In the sample representing group 2 (75%SLs) a dashed red square and a dashed blue square indicate the regions that lead to T_C1_ and T_C2_, respectively. (**b**) Temperature dependent Hall resistance (R_xy_) plot of a sample containing 75% SLs taken at 0.02 T. Shaded red and blue areas under the plot signify the proposed contribution for the T_C1_ and T_C2_ components. Inset: R_xy_ as a function of the magnetic field, B, measured at 10 K and 75 K.

A rationale for the presence of two T_C_ components in the samples is suggested if we consider the structural details of the samples. Figure 4a shows illustrative schematics of three samples representing the three groups described above, with %SLs of 20%, 75% and 95%. The proposed structures are consistent with TEM images obtained for our samples (Supporting Information Fig. S3). The sample with 20% SLs, shown on the left-hand panel of Fig. 4a, representative of group 1, is expected to have a single T_C_ value of 15-20 K, similar to T_C_ values reported by others for samples with few or single SLs ^13,16^ separated by QLs, and as seen in Fig. 1d. The sample with 95% SLs, shown in the right-hand panel of Fig. 4a, representative of group 3, would be expected to have higher T_C_ values, similar to the reported values for Mn-rich MnSb_2_Te_4_ samples containing Mn_Sb_ antisites^18^. Samples with 70–80% SLs (group 2) have a few single QLs randomly distributed through the mostly-SL containing structure, as illustrated in the middle panel in Fig. 4a. We propose that this distribution of single QLs within a mostly SL containing structure results in two distinct regions within the structure as marked with the red and blue dashed squares on the illustration. The magnetization could then be governed by two contributions, originating from the stacked SLs and from the QLs, respectively. The stacked SLs would be associated with T_C1_ and the QLs with T_C2_. Support for this proposal is described in the discussion that follows. In this discussion we do not discuss interlayer coupling between the QL and SL system.

As previously discussed^20^, a critical amount of Mn is necessary to initiate the SL growth and obtain the mixed QL:SL structures of (Sb_2_Te_3_)_1−x_(MnSb_2_Te_4_)_x_. This results in two types of Mn atoms: (1) Mn located in the center of the SL and (2) Mn which substitutes Sb, in SL and QL (Mn–Sb antisites). The latter will have an important effect on the Mn–Mn interaction. For stoichiometric MnSb_2_Te_4_ SL, while the intralayer Mn–Mn interaction is ferromagnetic (FM), the coupling between SLs is antiferromagnetic (AFM)^23^. An interlayer FM interaction is made possible in the presence of Mn–Sb antisites, favored by Mn–Te–Mn superexchange^15^ mechanism; for an antisite rate of 15–17%, T_C_ = 24–34 K has been observed in MnSb_2_Te_4_^15,19,24^. These temperatures are comparable to the T_C_ (or T_C1_) observed in our samples (See Arrot plots, Supporting Information Fig. S1): T_C_ varies slowly from 15 K in group 1 to about 20–30 K (possibly as high as 40 K) in group 3 when the SL density and the Mn-composition increase, which favors Mn–Sb substitution. We note that the SLs regions in the group 2 samples (red dashed square) are similar to the all-SL structure (right-hand panel of Fig. 4a), which exhibit T_C_ values as high as 30 or 40 K, as samples in Fig. 1f, close to what was observed for MnSb_2_Te_4_ single crystals^15^ or epilayers^18^.

For the QL contribution (T_C2_), it is known that alloys Sb_2−y_TM_y_Te_3_, where TM is a transition metal atom (V, Cr, Mn), present a ferromagnetic phase. This FM phase has been experimentally observed with high Curie temperatures: T_C_ = 177 K for TM = V and y = 0.35^25^; T_C_ = 190 K, for TM = Cr and y = 0.60^26^. For TM = Mn, a lower T_C_ has been measured for very small y, (T_C_ = 8.6 K and 17 K, for y = 0.02 and 0.03, respectively), and high y values have not been previously realized experimentally. Theoretically, this coupling has been explained by an exchange mechanism via TM-Te-TM bond, and the hybridization between the TM d states and the Te p states^27,28^. A possible in-plane coupling via carriers has also been discussed^29^. However, recent results on Mn-doped Bi_2_Te_3_ and BiSbTe_3_ have invalidated the latter mechanism ^30,31^ The TM-concentration dependence of T_C_ has also been theoretically predicted^29^. For TM = Mn, one expects T_C_ ≈ 40 K and 80 K for y = 0.25 and 0.50, respectively, the Mn–Mn coupling being smaller than for Cr and V. Thus, the high T_C2_ we observe could be attributed to highly Mn-doped QLs or Sb_2−y_Mn_y_Te_3_ alloys possibly formed under non-equilibrium MBE conditions, in a regime where a very high Mn flux is necessary to increase the SL density. According to theory^29^, for a T_C_ of 80 K a composition of Sb_1.5_Mn_0.5_Te_3_ would be needed. We have used the results of Table 1 to estimate the composition y (for the term Sb_2−y_Mn_y_Te_3_) of our QLs for the samples given in Fig. 3. For this, we assume a composition (Sb_2−y_Mn_y_Te_3_)_1−x_ (Mn(Sb_2−y_Mn_y_)Te_4_)_x_ for our group 2 samples (~ 75% SLs), which means that we neglect Sb_Mn_ antisites in the SLs and a fraction (y) of the Sb sites in both the QL and SL are occupied by Mn (Mn_Sb_ antisites). We then solve for y:

1 $$ y = \chi_{Mn} (5 + 2x) - x $$

where χ_Mn_ is the Mn fraction measured by EDS and x is the fraction of SLs in the structure. The values of y obtained for the samples in Fig. 3 are listed in Table 1. A plot of the T_C_ as a function of y is given in Fig. 3c, with theoretically predicted values^29^ of T_C_ also shown for comparison. Very close agreement between the theoretically predicted T_C_ values and the estimated values of y in our samples is observed, supporting the plausibility of our proposed mechanism. At low SL density, grown with lower Mn BEP rations, with low Mn concentration (group 1), the QLs are either paramagnetic or with a very low T_C2_, while at very high SL density (group 3), the QL are barely contributing to the magnetization due to their very low number.

The concept of two regions in the sample with different T_C_ values is further illustrated in Fig. 4b, which shows the T_C_ plot for a sample with x = 0.75 (75% SLs) grown with a 0.10 Mn BEP ratio. This plot can be viewed as the sum of two independent T_C_ plots: the T_C1_ plot marked by the red shaded area corresponding to the “all-SL” regions of the structure, and the T_C2_ plot marked by the blue shaded area corresponding to the QLs. At low temperatures the T_C1_ dominates since it represents a larger volume of the sample, but as the temperature increases above T_C1_, the T_C2_ component dominates appearing as a weaker tail, due to its lower volume and possibly its lower electrical resistance.

**Figure 5:**
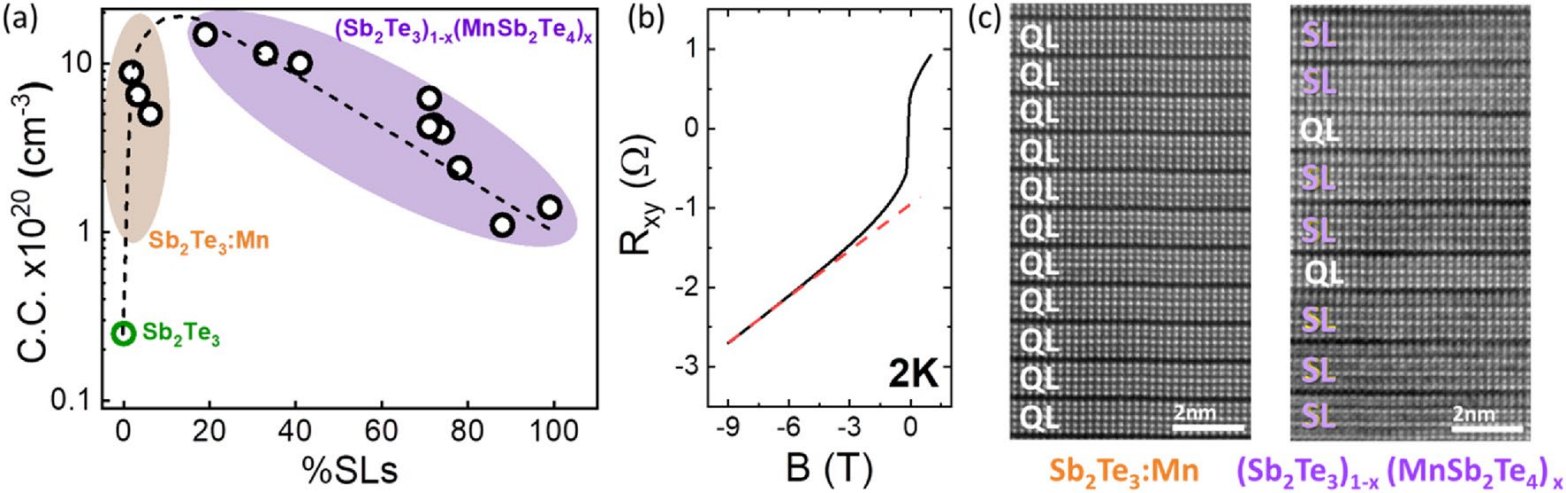
Carrier density for the (Sb_2_Te_3_)_1−x_(MnSb_2_Te_4_)_x_ samples. (**a**) Measured carrier density (C.C.) for a selection of samples including pure Sb_2_Te_3_ (green circle), Mn doped Sb_2_Te_3_ (orange shaded area) and (Sb_2_Te_3_)_1−x_(MnSb_2_Te_4_)_x_ (purple shaded area) with dashed line drawn to express the trend of the change in carrier density of the samples. Carrier density for all the samples was measured at 2 K except for Sb_2_Te_3_ (green circle), which was measured at 10 K (**b**) Field dependent Hall resistance (R_xy_) measurement of a sample taken at 2 K up to 9 T. Dashed red line drawn to show the linear part of the plot. (**c**) High-angle HAADF STEM images of a Mn doped Sb_2_Te_3_ sample and a representative sample containing QL and SLs. The QLs and SLs are marked by white and purple labels, respectively.

The requirement that an excess of Mn is needed in the samples for FM behavior and to achieve high T_C_ values suggests that an understanding of the role of Mn in the crystal as an electrical dopant is also needed. At low concentrations, substitutional Mn in Sb sites is expected to be a p-type dopant in Sb_2_Te_3_^21^. We have investigated the bulk background doping in our samples as a function of the Mn flux ratio and the %SL. Figure 5a shows the carrier density of a selection of samples with %SLs varying between 0 and 100%. Due to the internal magnetic moments of our samples, a high magnetic field was needed for the Hall Effect measurements to calculate accurately the carrier density. At high enough field, the field dependent Hall resistance plot returns to linearity, from which the carrier density is obtained, as illustrated in Fig. 5b. Hall Effect was measured with an applied magnetic field of 5–9 T as needed to ensure linearity of the R_xy_. Figure 5c shows representative TEM images of samples with compositions within the orange (Mn doped Sb_2_Te_3_ samples) and purple shaded areas [samples with formula (Sb_2_Te_3_)_1−x_(MnSb_2_Te_4_)_x_] of Fig. 5a. All the samples have higher carrier density (C.C.) than a reference sample of undoped Sb_2_Te_3_ (green circle) of 2.4 × 10^19^ cm^−3^, consistent with the p-type doping character of Mn impurities in Sb_2_Te_3_. As Mn is added initially at low levels, too low for SLs to form, the Mn doped Sb_2_Te_3_ samples (Sb_2_Te_3_:Mn) show no change in composition (i.e., no alloy formation). However, a high carrier density is measured, rapidly increasing with the Mn BEP used during growth by more than one order of magnitude, reaching a maximum doping level of ~ 2 × 10^21^ cm^−3^. This confirms that Mn is a p-type electrical dopant for Sb_2_Te_3_. As was noted in Fig. 1c, at these low Mn content levels, the Sb_2_Te_3_:Mn samples do not show hysteresis (i.e., they are not FM). As soon as the Mn BEP ratio is high enough so that some SLs form, the carrier density level of the structure starts to drop and, as the proportion of QLs in the structure decreases, the carrier density for the structure also decreases, reaching a value of ~ 1 × 10^20^ cm^−3^ for the samples with close to 100% SLs (0% QLs). This behavior suggests that during MBE growth of (Sb_2_Te_3_)_1−x_(MnSb_2_Te_4_)_x_ structures by self-assembly, Mn incorporates in Sb_2_Te_3_ as a substitutional impurity until sufficient Mn is provided, at which point SLs can begin to form by the further incorporation of Mn as a structural element (central atom of the SL) at higher Mn flux ratios. The amount of substitutional Mn in the SLs is harder to predict from this experiment, but we can infer, from the decreasing carrier density with the increasing %SLs, that the SLs themselves have a lower carrier density than the QLs, and the carrier density of the mixed QL:SL structure is dominated by the fraction of QLs in the structure. The direct dependence of the carrier density on the %SLs in these structures and the absence of correlation between T_C_ and C.C. for the samples in Table 1 suggest that the magnetic coupling mechanism responsible for the high T_C_ values observed at 70–80%SL is not carrier density dependent. The presence of a T_C2_ for 70–80%SLs suggests that at sufficiently high Mn BEP ratios during growth (> 0.07) a very large excess Mn may incorporate into the QLs, producing Sb_2−y_Mn_y_Te_3_ alloys, which are FM materials with high T_C_ values. The generally high carrier densities of these structures as grown by MBE suggest that modified structures may be needed to reduce the bulk electrical doping of the structures, as desired for topological applications. The use (Sb,Bi)_2_Te_3_^32^ alloys may be a promising alternative.

An important aspect of interest for these materials is their topological nature. Physical characterization studies including scanning tunneling microscopy (STM), or angle resolved photoelectron spectroscopy (ARPES) investigations combined with theoretical modelling studies, which are beyond the scope of this paper, would be useful to elucidate the band structure modifications produced by multiple QL:SL layered structures and the incorporation of the magnetic atoms in those structures that lead to the observed behavior. Such studies are particularly desirable, especially in view of the fact that ARPES has already evidenced a magnetic gap opening of 17 meV in 100%SL MnSb_2_Te_4_^18^. Further, topological surface states have also been shown in Mn doped Sb_2_Te_3_^33^ and a 90 meV gap opening was observed in self-organized mixed SL:QL such as (Bi_2_Te_3_)_1−x_(MnBi_2_Te_4_)_x_ heterostructures ^33^.

## Conclusions

We have investigated the magnetic properties of (Sb_2_Te_3_)_1−x_(MnSb_2_Te_4_)_x_ structures ranging in composition between Sb_2_Te_3_ (x = 0) and MnSb_2_Te_4_ (x = 1) which were previously grown by a self-assembly process in MBE by varying the Mn to Sb BEP ratio during growth. All the samples with more than a few SLs show FM behavior, likely due to magnetic disorder due to excess Mn in our samples. Three different T_C_ behaviors were observed depending on the value of x. Samples with x less than 0.7 (group 1) and samples with x greater than 0.9 (group 3) are described by a single T_C_ value of 15–20 K for group 1 and 20–30 K for group 3. These T_C_ values are consistent with reports of FM MnSb_2_Te_4_ by others^16–18^. A new behavior was observed for samples with intermediate values of x between 0.7 and 0.8. These samples exhibit a behavior consistent with having two Tc components, a T_C1_ value of ~ 23 K, and a higher T_C2_ value as high as 85 K in some samples. The highest values of T_C2_ are obtained for samples with x = 0.7–0.8 that were grown with high Mn BEP ratios, suggesting that excess Mn is important to enhance the high T_C2_ component. These T_C2_ values are the highest T_C_ values reported to date for these materials, nearly double the highest reported Tc values^18^. Remanent magnetization measurements using a SQUID magnetometer confirm the validity of these high T_C_ regimes. The high T_C2_ values were also verified by field dependent Hall resistance measurements done at high temperatures, which show hysteresis at temperatures as high as 80 K. Considering the structural distribution of SLs and QLs in the samples with 70–80%SLs, we propose that there are two distinct regions within the structures of these samples, each giving rise to a different T_C_ value. One region contains only SLs and gives rise to the T_C1_ component, while the other contains highly Mn doped QLs and are likely responsible for the high temperature T_C2_ component, in presence of a high Mn concentration, as predicted for Sb_2−y_Mn_y_Te_3_ alloys^29^. A study of the carrier density of the samples shows that as Mn is added during MBE growth, at very low Mn fluxes the Mn incorporates as a p-type dopant of Sb_2_Te_3_ increasing its carrier density up to 2 × 10^21^ cm^−3^. Once SLs start to form the carrier density of the samples decreases as the %SLs in the structure increases, reaching a value of 1 × 10^20^ cm^−3^ for samples with near 100% SLs. From this observation we conclude that a modification of the structures would be needed to lower the bulk carrier density in these materials, as needed for the observation of exotic physical phenomena expected of these topological materials. The results presented provide experimental evidence for high temperature ferromagnetism in these materials, paving the way for demonstration of practical applications of these novel quantum materials. They also provide essential information that may lead to the “on-demand” controlled growth of magnetic topological materials structures with desired optimized magnetic properties.

## Methods

All samples were grown in a Riber 2300P MBE system with base pressure of 3–5 × 10^–10^ Torr. The chamber is equipped with reflection high-energy electron diffraction (RHEED) for in-situ growth monitoring, and the samples were deposited on epi-ready c-plane (0001) sapphire substrates. High purity elemental 6N Sb, Te and 5N8 Mn sources were used. The details of the MBE growth have been previously reported^20^.

Scanning transmission electron microscope (STEM) images were performed (EAG Laboratories) using a Hitachi HD-2700 Spherical Aberration Corrected Scanning-TEM system. Carrier density and field and temperature dependent measurements were performed in a 14 T Quantum Design physical property measurement system (PPMS) in 1 mTorr (at low temperature) of He gas or in a Lakeshore 7600 electromagnet system. Electrical contacts in the van der Pauw configuration were made with indium bonded on the edge of the thin film.

Magnetization measurements were performed with a superconducting quantum device (SQUID) magnetometer (Quantum Design MPMS—XL). The rapid scan option (rso) of the MPMS-XL was used, giving the opportunity to acquire data at a high speed (0.5 Hz) and average on 5 measurements.

## Supplementary Information

Supplementary Information.

## Data Availability

The datasets used and/or analyzed during the current study are available from the corresponding author on reasonable request.
