# Supplementary material for: High Curie temperature ferromagnetic structures of (Sb2Te3)1−x(MnSb2Te4)x with x = 0.7–0.8
Source: Sci Rep. 2023 May 6;13:7381. doi: 10.1038/s41598-023-34585-y (PMC10164192; doi:10.1038/s41598-023-34585-y)
Supplement: Supplementary file 1 — Supplementary Information. [file 41598_2023_34585_MOESM1_ESM.docx]

High Curie Temperature Ferromagnetic Structures of (Sb_2_Te_3_)_1-x_(MnSb_2_Te_4_)_x_ with x = 0.7-0.8 ​

Ido Levy,^1,2^ Candice Forrester,^1,2^ Xiaxin Ding,^3^ Christophe Testelin,^4^ Lia Krusin-Elbaum,^3,5^ and Maria C. Tamargo*,^1,2^

^1^Department of Chemistry, The City College of New York, NY 10031

^2^Ph.D. Program in Chemistry, The Graduate Center of the City University of New York, NY 10016

^3^Department of Physics, The City College of New York, NY 10031

^4^Sorbonne Université, CNRS, Institut des NanoSciences de Paris, F-75005 Paris, France

^5^Ph.D. Program in Physics, The Graduate Center of the City University of New York, NY 10016

*Corresponding author: [mtamargo@ccny.cuny.edu](mailto:mtamargo@ccny.cuny.edu)

Supporting Information Section

**Abstract:**

The Supporting Information section contains a Section entitled:

Section 1. Description of the Molecular Beam Epitaxy growth conditions

and three figures:

Figure S1. Arrott plots of the magnetization data for samples from the three groups.

Figure S2. Hall resistance and derivative plots for two samples from group 2.

Figure S3. High-resolution Transmission Electron Microscopy images for representative samples from the three groups.

1. **Description of the Molecular Beam Epitaxy Growth Conditions.**

As described in Reference 20 of the text,^1^ all samples were grown in a molecular beam epitaxy (MBE) system, Riber 2300P, with a base pressure of 3-5 × 10^−10^ Torr, equipped with reflection high-energy electron diffraction (RHEED) for *in-situ* growth monitoring, on epi-ready c-plane (0001) sapphire substrates. The substrates were heated under vacuum, in order to remove impurities from the surface, to 600 °C for 1 h prior to growth. High-purity 6N antimony (Sb), tellurium (Te), and 5N8 manganese (Mn) sources were used and their fluxes were achieved using a Riber double zone cell for Sb and single-zone Knudsen cells for Mn and Te. An ion gauge placed in the position of the substrate prior to growth was used to determine the fluxes, measured as beam equivalent pressure (BEP). The Mn BEP ratio, BEP(Mn)/[BEP(Mn) + BEP(Sb)], is proportional to the Mn flux fraction, and was used to control the Mn content in the layer during growth by varying it between 0.00 and 0.11. The growth was performed under excess Te flux and the samples were grown via a two-step growth method consisting of an initial deposition of a thin (3−5 nm) low-temperature buffer (LTB) for ∼3 to 5 min of Sb_2_Te_3_ layer, at 200 °C. The growth was then stopped, while keeping constant Te flux at the sample surface, and the substrate temperature was raised to the growth temperature of which varied between 245 and 275°C. The Mn-containing layer was then grown for 1−2 h, using the Mn, Sb, and Te sources simultaneously. A Te/Sb BEP ratio was kept between 20 and 30, ensuring excess Te during growth. All samples were grown using a similar Sb flux, and the composition was controlled by adjusting the Mn cell temperature (hence changing the Mn flux). The growth rates for the samples were between 0.4 and 1.2 nm/min depending on the Mn flux used.

**S1.**

**
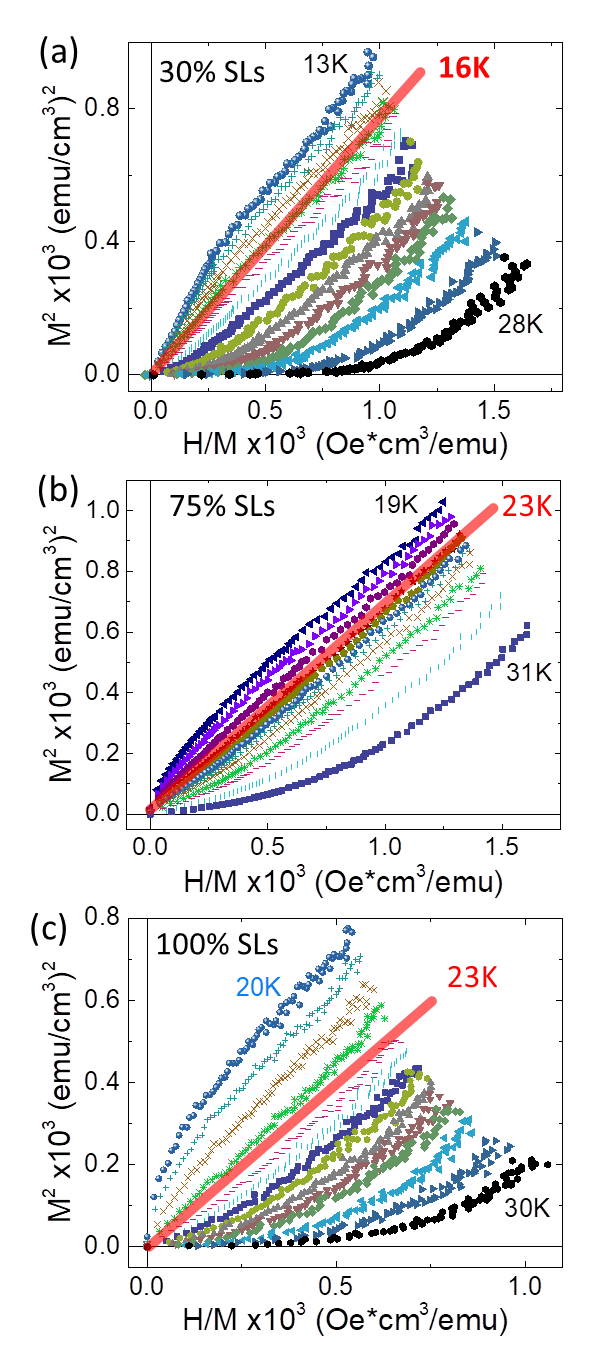
**

**Figure S1:** Arrott plots of the magnetization data for samples from the three groups, assuming mean field theory conditions. (a) Sample from group 1 with 30% septuple layers (SLs) (x=0.3) showing a T_C_ value of 16K. (b) Sample from group 2 with 75% SLs (x=0.75) showing a T_C1_ value of 23K. (a) Sample from group 3 with 100% SLs (x=1.0) showing a T_C_ value of 23K.

**S2.**


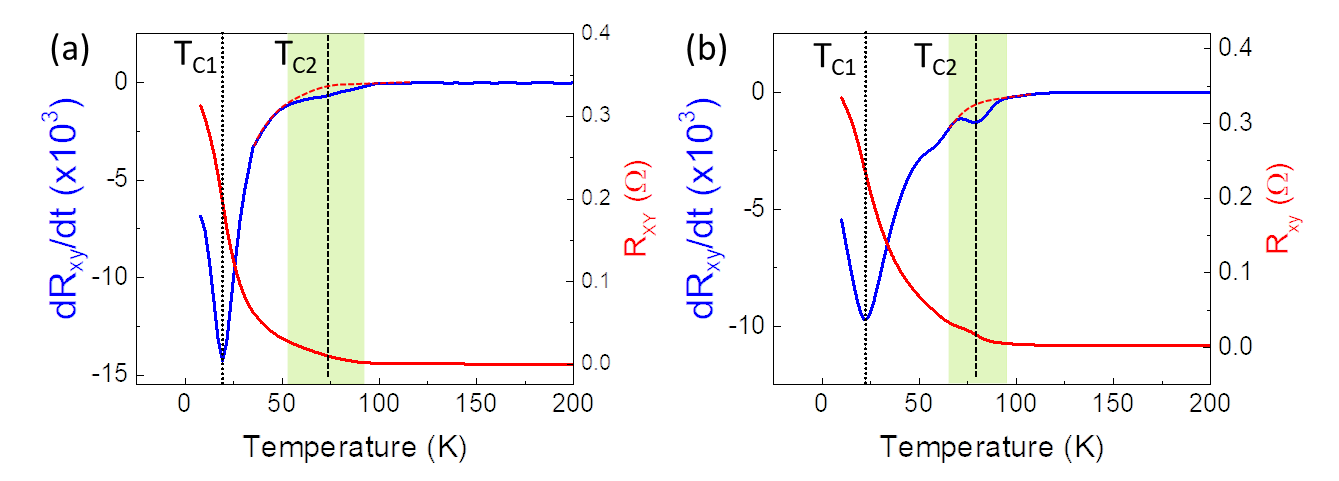


**Figure S2.** Hall resistance and derivative plots for two samples from group 2. (a) Sample with 75% SLs showing two T_C_ values, T_C1_ at 23K and T_C2_ at 75K. (a) Sample with 78% SLs showing two T_C_ values, T_C1_ at 24K and T_C2_ at 80K.

**S3.**


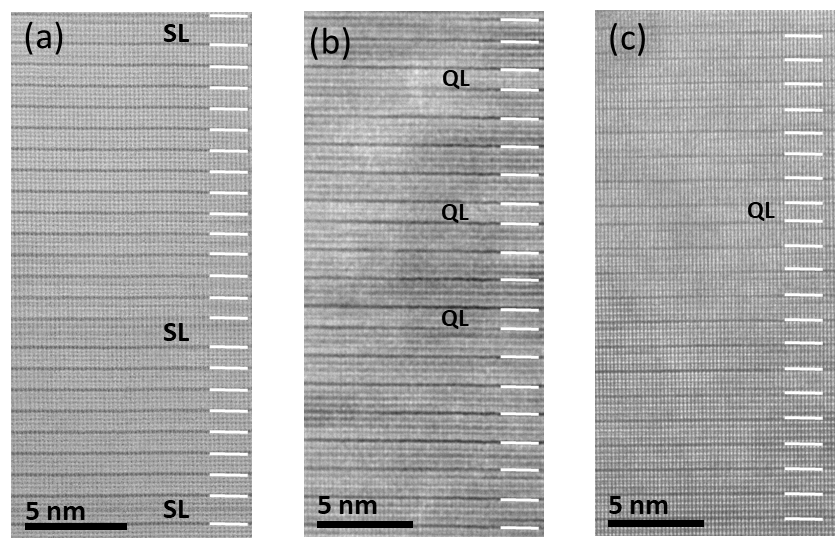


**Figure S3.** High-resolution Transmission Electron Microscopy (HR-TEM) images for representative samples from the three groups. (a) Sample from group 1 containing 13% SLs. (b) Sample from group 2 containing 75% SLs. (c) Sample from group 3 containing 90% SLs.

References:

1. Levy, I. *et al.* Compositional Control and Optimization of Molecular Beam Epitaxial Growth of (Sb_2_Te_3_)_1−x_(MnSb_2_Te_4_)_x_ Magnetic Topological Insulators. *Cryst. Growth Des.* **22**, 3007-3015 (2022) DOI:10.1021/acs.cgd.1c01453.
